# Care of Children’s Teeth

**Published:** 1899-02

**Authors:** 


### EDITORIAL.

### Care of Children's Teeth.

This subject is now receiving more attention than ever before from the fact that a knowledge of the value of the teeth and of the almost universal prevalence of disease of the teeth, and that proper attention will in a large measure lessen this disease, is inducing the people generally to look more carefully into the subject.

This is a question to which dentists should give more attention than is usually done. Attention in not only preventing and remedying the affections of the teeth, which is so very general, but they should also endeavor by all reasonable means to give proper information on the subject to all having the care of children.

The introduction of information on this subject in some form or other into the public schools, and especially to the attention of teachers and superintendents of schools, is now being more agitated than ever before. It is suggested that instruction pertaining to the teeth may be introduced into the regular school work, and the more simple principles of preserving and caring for the teeth given and so impressed upon the minds of all the children as to result in great good. The best manner of accomplishing this is now receiving special attention by some of our best informed dentists, and some educators also have the subject under consideration.

The introduction of the subject of the teeth, their care and treatment as a department in an elementary work for the public school has been suggested as feasible. In addition to this it has been suggested that teachers be thoroughly prepared for giving instruction in regard to the teeth and their care. It is also suggested that competent persons be designated whose duty it shall be to visit schools and give instruction in the way of lectures, etc.

We will here suggest that the preparation of a brief treatise on the subject of the teeth, their diseases and care, prepared for and put into the hands of all teachers of the young, with a view of having the information utilized in their school work, would likely in a large measure secure attention.

This subject could also be considered in teachers' institutes with very great benefit in the way of essays and lectures of such character as to give the needed information to teachers, and serve as a stimulus and arouse proper interest.

Whether any or all of these would be feasible can only be determined by experiment. About one thing, however, there is no question—that information for the public on this subject is greatly needed and would ultimately be of immense value.

